# Oncologic Outcomes of Young Breast Cancer Patients According to Tumor Biology

**DOI:** 10.3390/cancers17081333

**Published:** 2025-04-15

**Authors:** Marijana Henzler, Kay C. Willborn, Wolfgang Janni, Jens Huober, Stefan Lukac, Burkhard Otremba, Wenjie Shi, Luz Angela Torres-de la Roche, Rudy Leon De Wilde

**Affiliations:** 1 University Hospital for Gynecology, Pius Hospital, University Medicine Oldenburg, Georgstrasse 12, 26121 Oldenburg, Germany; marijana.henzler@pius-hospital.de (M.H.); rudy-leon.dewilde@pius-hospital.de (R.L.D.W.); 2 University Hospital for Medical Radiation Physics, Pius Hospital, University Medicine Oldenburg, Carl von Ossietzky University Oldenburg, 26121 Oldenburg, Germany; kay.willborn@pius-hospital.de; 3 Department for Obstetrics and Gynecology, University Hospital Ulm, 89070 Ulm, Germany; wolfgang.janni@uniklinik-ulm.de (W.J.); stefan.lukac@uniklinik-ulm.de (S.L.); 4 Breast Cancer Center St. Gallen, Kantonsspital St. Gallen, 9007 St. Gallen, Switzerland; jens.huober@kssg.ch; 5 Oncological Practice Oldenburg, 26121 Oldenburg, Germany; otremba@onkopraxis-oldenburg.de; 6 Department of Breast Surgery, EUSOMA Certified Breast Center, Guilin TCM Hospital of China, Guilin 540102, China; wenjie.shi@uni-oldenburg.de

**Keywords:** breast cancer, tumor biology, young women, oncological outcomes

## Abstract

Background/Objectives: Young women frequently present with more aggressive breast cancer tumors. This retrospective study analyzed the oncological outcomes of patients under the age of 40 according to the tumor biology. Methods: Group comparisons were performed via the log-rank test. Recurrence and survival rates are presented according to the Kaplan–Meier method. Results: In total, 88 women (mean age 36) were included, but two presented with bilateral cancer, resulting in 90 tumors. Triple-negative carcinoma was most common, with 26.7% (n = 24); 11.1% (n = 10) were luminal A; 23.3% (n = 21) were luminal B HER2-negative; 15.6% (n = 14) were luminal B HER2-positive; and 6.7% (n = 6) were HER2-positive (non-luminal). Moreover, 26.1% (n = 23) of patients experienced recurrence (mean 40 months), with the highest recurrence rate in the HER2-positive (50%) and triple-negative (30.4%) groups. The 3- and 5-year recurrence-free survival rates were 84.9% and 77.3%, and the overall survival rates were 93.1% and 90.3%, respectively. No statistically significant differences in oncological outcomes were observed (*p* = 0.164). Conclusions: The results show that young women tend to have triple-negative and fast-growing breast carcinomas, with worse overall survival in the triple-negative group. More research is needed on the pathomechanisms of breast cancer development in young women, especially those leading to disease progression and resistance to therapy.

## Simple Summary

Young women often have more aggressive breast cancer tumours. This study looked at how patients under 40 years old with different types of breast cancer reacted to treatment and how often the cancer came back. 88 women took part in the study (average age 36), two of them had cancer in both breasts, therefore 90 tumours were analyzed. The most common type of cancer was triple-negative (26.7%), followed by luminal B HER2-negative (23.3%), HER2-positive or non-luminal (15.6%) and Luminal A (11.1%). In 26.1% of patients (23 out of 87), the cancer reappeared after an average of 40 months, most often in patients with HER2-positive (50%) and triple-negative (30.4%) cancer. The chances of the cancer coming back again after three and five years were 84.9% and 77.3%, respectively. The survival rates were 93.1% and 90.3%, respectively. There were no big differences in how well the cancer was treated. The results show that young women tend to have triple negative and fast-growing breast cancers, with worse overall survival in the triple negative group. We need to do more research to understand how breast cancer develops in young women, especially how the disease progresses and becomes resistant to therapy.

## 1. Introduction

Breast cancer (BC) is the most commonly diagnosed cancer worldwide. Based on the current incidence rates, approximately one in eight women will develop breast cancer during her lifetime [1,2,3]. With around 71,375 new cases per year, BC is by far the most common cancer among women in Germany; more than 6000 are in situ tumors [4]. Despite the relatively low incidence in women before age 40 (4% of all breast cancers), it is the most common new cancer in this patient population (incidence 115/100,000 women) and is considered the most common cause of oncological death in young women (26% of all cancer-related deaths) [2,3,4]. Young women tend to have larger breast tumors, with more aggressive phenotypes and more advanced stages, compared to older women [5]. Studies show that more than 90% of young patients present with symptoms and up to 50% of patients are node-positive at pathological diagnosis [5,6]. A lack of oncological awareness among physicians treating young women with breast lesions, such as during pregnancy, postpartum, or lactation, could lead to delayed diagnoses, affecting the prognosis of the disease [7,8].

BC generally exhibits a more aggressive tumor biology in young patients. Exemplary studies for this patient population have documented that the proliferation marker Ki67 increases inversely proportionally to the patient’s age; G3 carcinomas are present in 60%, triple-negative carcinomas (TNBC) in 20%, and HER2-positive (non-luminal) carcinomas in 25% of cases. The proportion of the basal-like subtype is significantly higher and the proportion of the luminal A subtype is significantly lower than in women older than 50 years [9,10]. In addition, younger women with luminal tumors appear to have a less favorable outcome when compared to older patients [7,8,9,10,11,12,13]. Young patients are almost three times more likely to have BRCA1/BRCA2 mutations [7]. Aggressive subtypes, such as TNBC and HER2-positive disease, are more prevalent in this age group compared to the overall population [13]. Despite these differences, little research has focused on the biology of these tumors with the goal of improving the prognosis and developing potential treatment strategies for young patients [9,14,15]. Therefore, this study analyzed the oncological outcomes of young breast cancer patients according to the tumor biology.

## 2. Method

This was a monocentric, retrospective, comparative, descriptive, single-cohort study, including only young women diagnosed with BC and treated at the University Hospital for Gynecology, Pius Hospital Oldenburg, between January 2010 and December 2019. Cases were selected from the database of the Tumor Documentation Bureau of the hospital, according to the International Classification of Diseases (ICD-10). Cases that fulfilled the following inclusion criteria were selected for the final analysis: adult women younger than 40 years old (18 to 39 years) with BC, primary or metastatic, receiving treatment at the Pius Hospital during the defined study period. Cases with a personal history of any other invasive cancer, or patients who, at the time of treatment initiation, signed an explicit non-consent form regarding data analysis, were excluded.

An analysis of patient characteristics and oncological outcomes was performed. Demographic characteristics included age, height, weight, body mass index (BMI), menopausal status (pre- or postmenopausal), gravidity, parity, and metabolic abnormalities. Tumor characteristics included the stage at diagnosis, tumor histological grade, receptor status, proliferation marker (Ki67), and tumor biology subtype, according to the World Health Organization and Union for International Cancer Control [16,17]. Oncological outcomes included the recurrence rate, recurrence-free survival, and overall survival. Categorical variables are presented as counts and percentages. Continuous variables are presented as means and standard deviations or as medians and ranges, as appropriate.

In order to analyze the oncological outcomes according to the tumor biology, cases were divided into five groups according to the subtype classification of the German S3 Breast Cancer Guidelines [18]: luminal A (Ki67 < 14%), luminal B HER2-negative (Ki67 ≥ 14%), luminal B HER2-positive, HER2-positive (non-luminal), and triple-negative. In only one patient was an adenoid cystic carcinoma diagnosed. For this reason, it will be discussed individually, rather than categorized as a separate group. With regard to cases in which the Ki67% values were unknown, they were excluded from the comparative analysis because both luminal A and luminal B HER2-negative tumors were present in this group and differentiation was not possible without a Ki67 value.

The general characteristics of the study population were analyzed based on the total number of patients who met the inclusion criteria (n = 88). However, in anticipation of the occurrence of patients with bilateral cancer, the denominator for the statistical analysis of the tumor stage and characteristics was calculated according to the total number of tumors (n = 90) diagnosed in the entire population. Furthermore, the total number of patients was considered in order to facilitate comparisons between the groups with regard to survival and recurrence. Survival data were assessed via the log-rank test with a two-sided significance level of 5%. Recurrence and 3- and 5-year survival rates are presented in a curve according to Kaplan and Meier.

## 3. Results

**Figure 1:**
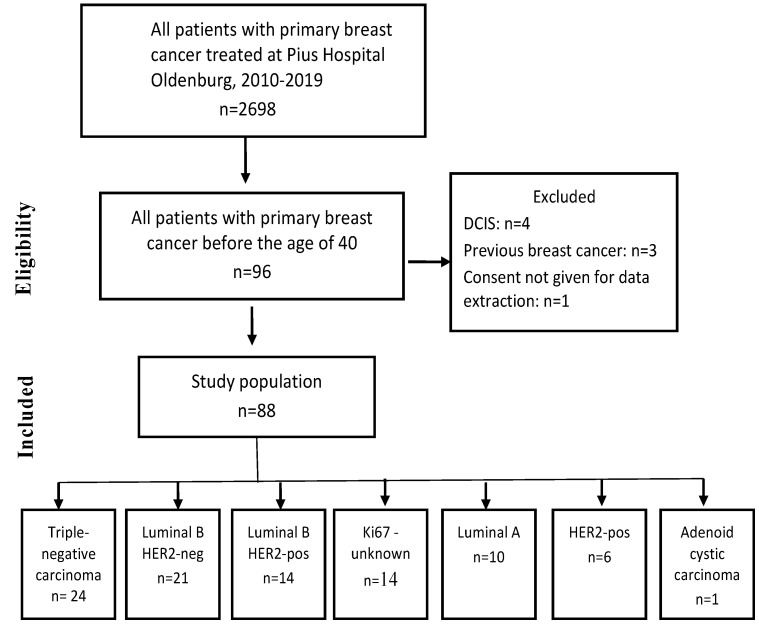
Selection of patients with primary breast cancer before the age of 40.

Initially, 2698 patients with primary BC, treated between January 2010 and December 2019, were identified in the hospital database. After a review of the inclusion and exclusion criteria, a cohort of 88 patients, two of whom had invasive breast carcinoma on both sides at initial diagnosis, was included in the analysis (Figure 1).

At the time of initial diagnosis (Table 1), all 88 patients were premenopausal, with the youngest patient being 23 years old and the oldest 39 years old (mean 36 y). According to the BMI classification, 2.3% (n = 2) of the women were underweight, 56.8% (n = 50) were of a normal weight, 29.5% (n = 26) were overweight, and obesity was present in 11.3% (n = 10) of cases. Metabolic parameters such as fasting blood glucose, triglycerides, HDL, and LDL showed mean values within the normal range. Only the mean cholesterol value of 192 mg/dl was slightly above the normal value; hypertension was present in 6.8% (n = 6) of cases; and 68.2% (n = 60) were previously pregnant. Moreover, 43.2% (n = 38) had a positive BC family history, of which 36.4% (n = 32) had one, 6.8% (n = 6) had two, and 2.3% (n = 2) had three relatives with BC. Among the 21 examined patients eligible for the BRCA mutation test [19], 42.9% were positive, corresponding to 10.2% (n = 9) of the cohort. The left and right breasts were equally affected by cancer (48.9% each), but two women presented (2.3%) with bilateral disease at the time of initial diagnosis. The mean follow-up period was 80.5 months (range: 4–148 months), with 92.1% of patients followed for more than 3 years.

The characteristics of all breast cancers initially diagnosed in the cohort (n = 90) are presented in Table 2a. The clinical tumor size (cT) was used in cases that underwent neoadjuvant therapy, and the pathological tumor (pT) size was used for those that received an adjuvant therapy. The most common tumor size was T1c, with 47.8% (n = 43) of cases, followed by T2 with 34.5% (n = 31), T1b with 12.2% (n = 11), and T3 with 3.3% (n = 3). The lowest number, 2.2% (n = 2), was observed in the T1a category. No patient had a T4 tumor. In patients who received neoadjuvant chemotherapy (n = 21), ultrasound and MRI findings before chemotherapy were considered for the initial cT staging. From this group, 54.5% (n = 12) of cases achieved pathological complete remission, and, in the other 45.5% (n = 10), the mean size was 6.6 ± 5.4 mm. Among the patients that received an adjuvant therapy (n = 67), the postoperative histological examination of the tumor size revealed a mean pT size of 17.9 mm (range 3 to 68 mm).

The majority of cases, namely 62.2% (n = 56), showed no lymph node involvement; 25.6% (n = 23) were classified as N1, 7.8% (n = 7) as N2, and 4.4% (n = 4) of cases as N3. Distant metastasis at diagnosis was present in 3.3% (n = 3) of cases. Lymphatic vessel and venous invasion were reported in 27.8% (n = 25) and 3.3% (n = 3) of cases, respectively, but, in 15.5% (n = 14), these parameters were not described.

Regarding the biology of the 90 tumors at the time of diagnosis, most were carcinomas, at 96.7% (n = 87), and had a “non-specific type (NST)”, formerly the invasive–ductal tumor type. Other histological tumor types occurred only sporadically (Table 2b). Moreover, 11.1% (n = 10) of the tumors were luminal A, 23.3% (n = 21) were luminal B HER2-negative, and 15.6% (n = 14) were luminal B HER2-positive tumors. The triple-negative subtype was most common, with 26.7% (n = 24). In contrast, the HER2-positive group had the lowest number, with 6.7% (n = 6). Among the two cases with bilateral breast carcinomas, one patient had a triple-negative carcinoma on the right side and a HER2-positive carcinoma on the left side. The other patient had a luminal B HER2-positive tumor in the right breast and a luminal B HER2-negative tumor in the left. In 14 tumors, no differentiation between luminal A and luminal B HER2-negative could be achieved in the absence of a Ki67 report. One case of an adenoid cystic carcinoma, a unique histological type with low malignant potential [16], was reported.

Most tumors (64.4%; n = 58) were classified as the G3 grade, 32.2% (n = 29) as the G2 grade, and 2.2% (n = 2) as the G1 grade. The hormone receptor status was positive in the majority of cases, ER+ in 65.5% (n = 59), and PR+ in 62.2% (n = 56). A positive HER2-neu status was present in 24.4% (n = 22) of tumors. In this study, the proliferation rate of Ki67, as a marker of the growth rate, was divided into two categories, with a cut-off value of 14, according to the St. Gallen International Expert Consensus on the Primary Therapy of Early Breast Cancer [20]: a low proliferation rate with a Ki67 rate lower than 14% and a high proliferation rate with a Ki67 rate higher than 14%. A low proliferation score was present in 12.2% (n = 11) of tumors, compared with 63.3% (n = 57) of tumors with a score greater than 14%. The mean value was 34.8% (range 4% to 85%); this value was not reported in 24.4% (n = 22) of cases because the determination of Ki67 only became an integral part of the histopathological examination in mid-2013.

All patients underwent guideline-based therapy after interdisciplinary consultation among the hospital’s tumor board. Neoadjuvant treatment was performed in 23.9% (n = 21) of cases and adjuvant treatment in 76.1% (n = 67). One patient did not receive surgical therapy due to rapid disease progression and death during neoadjuvant chemotherapy.

Among the surgical therapies performed, breast-conserving surgery was used to treat 73.9% (n = 65) of cases; 25% (n = 22) underwent mastectomies, including two patients with bilateral breast carcinomas, who required a bilateral mastectomy. A sentinel procedure was performed in 54.6% (n = 48) of cases, one of which was bilateral. Classic axillary dissection was performed in 44.3% (n = 39) of cases, one of which was bilateral. One patient with sonographically unremarkable axilla refused the recommended sentinel procedure. Patients with a confirmed BRCA mutation received appropriate interdisciplinary consultation; 55.6% (n = 5) of them opted for a prophylactic mastectomy and 33.3% (n = 3) for a prophylactic ovariectomy.

As mentioned, the majority of cases received chemotherapeutic agents as neoadjuvant or adjuvant therapies, but, in 12.5% (n = 11) of cases, this therapy was not indicated by the tumor board because of the tumor characteristics, the clinical condition of the patient, or non-consent. One patient declined the recommended adjuvant chemotherapy and another did not receive it due to a glucose-6-phosphate dehydrogenase deficiency. In most cases, combined cytostatic agents were used, with the choice of chemotherapy regimen based on the patient’s clinical condition, disease stage, and tumor biology. The two most frequently prescribed adjuvant therapies were (i) three cycles of fluorouracil, epirubicin, and cyclophosphamide followed by three cycles of docetaxel (n = 19) and (ii) four cycles of epirubicin and cyclophosphamide followed by 12 cycles of paclitaxel (n = 18). For the neoadjuvant regimen, the first four cycles of epirubicin and cyclophosphamide followed by paclitaxel alone or in combination with carboplatin were the most commonly prescribed (n = 10).

Endocrine therapy was provided to patients with a positive hormone receptor status, of whom 33.9% (n = 20) received tamoxifen alone, 59.3% (n = 35) received tamoxifen and GnRH analogs, and 5.1% (n = 3) received tamoxifen with a recommendation for conversion to aromatase inhibitors after 2 to 3 years. In one patient (1.7%), endocrine therapy with tamoxifen was omitted due to a heterozygous factor V Leiden mutation. Instead, a combination of anastrozole and goserelin was prescribed. The duration of use of the initial medication ranged from 2 to 10 years (mean 5.6 ± 2 years).

One year of antibody therapy was administered in those with a positive HER2-neu status with trastuzumab in 68.2% (n = 15) of patients, bevacizumab in only one patient (4.5%), and a combination of trastuzumab with pertuzumab in six cases (27.3%). Due to bone metastasis, one patient underwent bisphosphonate therapy with zolendronic acid.

Postoperatively, 70 cases (79.5%) underwent radiotherapy: 59 cases after breast conserving surgery and 11 after mastectomy. The most common indication for post-mastectomy radiation was the involvement of the axillary lymph nodes. The reasons for not receiving radiation included the following: two patients had undergone breast-conserving surgery but were in a metastatic situation, another patient experienced recurrence and rapid disease progression with the development of bone and lung metastases during adjuvant chemotherapy, and one patient declined the recommendation.

Regarding complications and adverse events, almost all postoperative complications were classified as Clavien–Dindo grade I [21]; only one patient developed a breast hematoma requiring revision on the left side after bilateral mastectomy. It was also observed that the chemotherapy administered was well tolerated by the majority of patients. According to the Common Terminology Criteria for Adverse Events (CTCAE) [22], severe adverse events (SAE) of grade 3 were reported in 15.9% (n = 10) of cases, including anemia, febrile neutropenia, infections, allergic reactions, fatigue, nausea, and emesis. No grade 4 or 5 adverse events were reported. In 16.0% (n = 12) of the cases, no information about the performed chemotherapy was available, because it was provided in other oncological centers. All hospital readmissions in this cohort were due to chemotherapy-associated SAE, with none of the patients requiring intensive care. The treatment regimen was modified, and there were no deaths resulting from SAE. In reference to adverse events that occurred during radiotherapy, most patients presented mild erythema or epitheliolysis. Less frequently, skin edema, fatigue, dermatitis, and skin dryness occurred. No SAE of grades 3 to 5 were documented.

**Figure 2:**
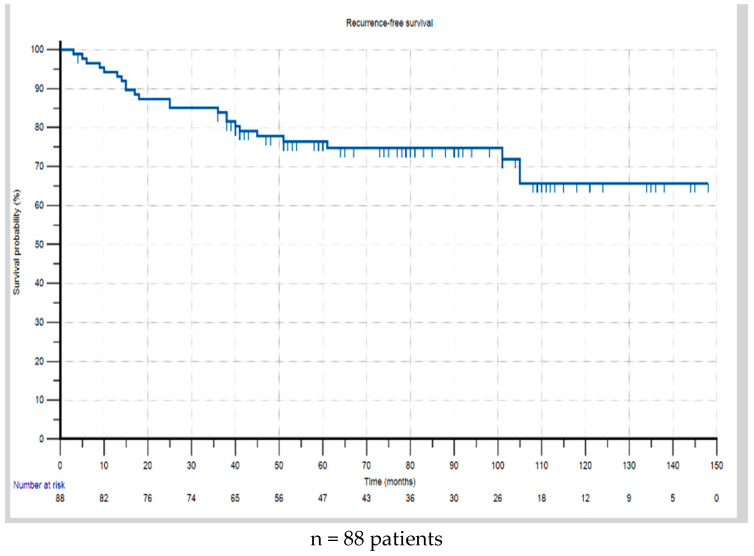
Recurrence-free survival from the time of diagnosis.

During the follow-up period, 26.1% (n = 23) of patients experienced recurrence after an average of 40.0 months (range: 3 to 147; SD ± 38.6 months). Of these, local recurrence was seen in 11.4% (n = 10) of cases, with metachronous metastasis in 9.1% (n = 8), i.e., metastasis developing after the completion of the initial curative treatment. In 5.7% (n = 5) of cases, concurrent local recurrence and metachronous metastasis occurred. The Kaplan–Meier analysis showed 3- and 5-year recurrence-free survival rates of 84.9% and 77.3%, respectively (Figure 2). It should be noted that 92.1% of the patients were followed up with for 3 years, with the shortest follow-up period being 4 months (mean: 80.5; range: 4–148 months).

According to the tumor biology, patients in the HER2-positive group had the highest rate of recurrence (50.0% of this group, n = 3), followed by the triple-negative group (30.4% of this group, n = 7), the luminal B HER2-positive group (28.6% of this group, n = 4), and the luminal B HER2-negative group (21.7% of this group, n = 5). The group with the fewest recurrences (n = 2) was the luminal A group. The Ki67-unknown group was excluded from the comparative analysis, as both luminal A and luminal B HER2-negative tumors were present in this group, and differentiation was not possible without a Ki67 value. The patient with an adenoid cystic carcinoma remained free of recurrence for the entire observation period of eight years.

**Figure 3:**
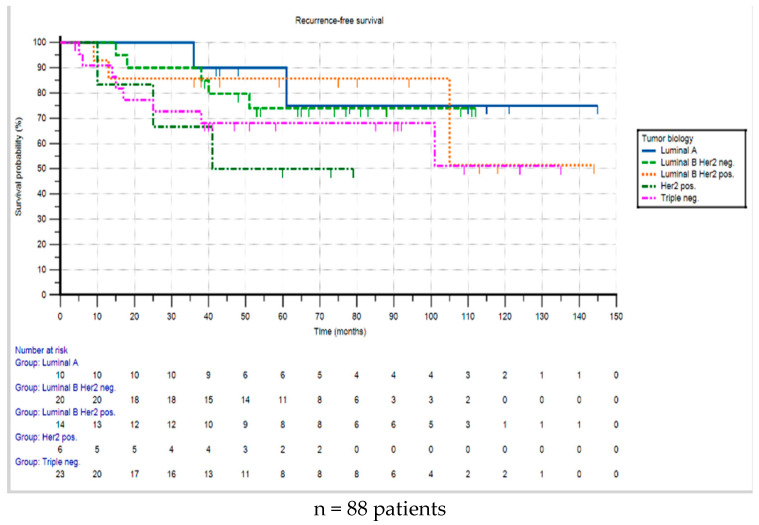
Recurrence-free survival from the time of diagnosis according to the tumor biology.

The Kaplan–Meier analysis revealed the following 3- and 5- year recurrence-free survival rates: for luminal A, 90% each; for luminal B HER2-neg, 90.0% and 74.0%; for luminal B HER2-pos, 85.7% each; for triple-negative, 76.2% and 71.4%; and for HER2-pos, 66.7% and 50.0%, respectively (Figure 3). The log-rank test showed no statistically significant difference in recurrence-free survival for the different subtypes (*p* = 0.494).

**Figure 4:**
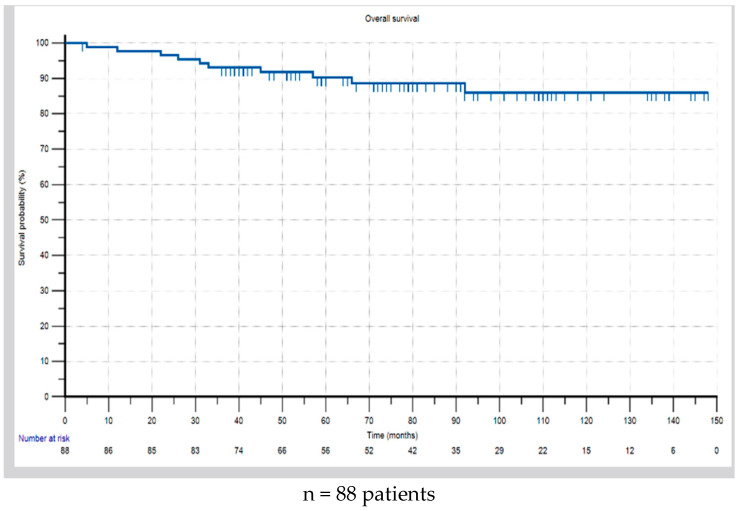
Overall survival of patients with breast cancer before the age of 40.

In regard to mortality, 10 deaths (11.4%) were reported, all of them due to the formation of distant metastases. For the overall study population, the Kaplan–Meier analysis showed 3- and 5-year survival rates of 93.1% and 90.3%, respectively (Figure 4). Half of the deceased patients (n = 5) had triple-negative carcinomas, three had luminal B HER2-negative tumors, one had a HER2-positive tumor, and one had a luminal B HER2-positive tumor. There were no deaths in the luminal A group. The patient with an adenoid cystic carcinoma was alive at the time of the last follow-up, eight years after the initial diagnosis.

**Figure 5:**
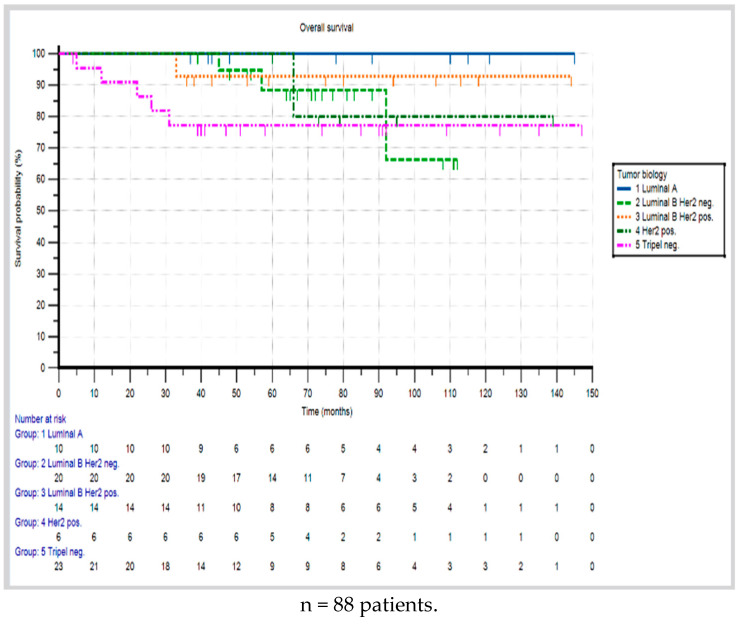
Overall survival rate of young breast cancer patients according to tumor biology.

Accordingly, the Kaplan–Meier analysis revealed the highest 3- and 5-year survival rates of 100% for the luminal A group. In comparison, luminal B HER2-positive tumors had slightly shorter survival periods, with 3- and 5-year survival rates of 92.9% each, and luminal B HER2-negative tumors had 100% and 88.4% survival rates, respectively. The triple-negative group had the lowest survival rates, with 3- and 5-year rates of 77.3% each (Figure 5). The log-rank test showed no statistically significant difference in overall survival for the different tumor subtype groups (*p* = 0.164).

## 4. Discussion

BC treatment has changed rapidly thanks to new advances in diagnostics, targeted therapies, and regular guideline updates, which enable patients to receive individualized treatment and improve their chances of disease-free survival. However, not all young women are benefiting from these advances, either because this population and/or their health caregivers are unaware of the increased incidence of the disease in this population or because high-risk patients are not being included in screening programs, leading to the later diagnosis of the tumor [5,7,8]. Consequently, women present with advanced stages of the disease, decreasing the possibility of conservative therapies or affecting their prognosis in terms of recurrence and survival. In fact, the proportion of breast malignancies among all new cancers in young women has increased rapidly worldwide since 1992, from 2% in those in their 20s to over 40% in those in their 40s [23]. In Germany, the incidence of breast cancer increased by >7.5% after the introduction of the screening program [24]; in fact, 29% of all new cancer cases occur in women under 40 [4]. Moreover, these women are three times more likely to have BRCA-1 or BRCA-2 mutations [7], HER2-positive tumors, and TNBC, i.e., a more aggressive disease.

This report presents the results of a retrospective, comparative analysis of young women treated at our breast cancer referral center between 2010 and 2019. During this decade, 88 out of 2698 patients (3.26%) with a primary tumor were younger than 40 years, with the youngest being 23 years old. Two of them presented with bilateral cancer, resulting in a total of 90 different tumors being analyzed. In the majority of cases, the hormone receptor status (ER/PR) was positive and the HER2-neu status was negative. TNBC was identified in almost one third of the cases, and the HER2-positive type was the least frequent. Most patients presented with pathological grade 3 fast-growing carcinomas and local disease, with one third of cases presenting lymph node involvement and two third with a Ki67 value greater than 14%. There were only three cases of distant metastatic disease at initial diagnosis.

Regarding the worldwide BC incidence, less than 0.5% of patients are younger than 20 years, which has remained stable in the last few decades; however, around 25% of cases are diagnosed in stages III or IV of the disease [23]. As expected, our youngest patient diagnosed with advanced regional TNBC (grade 3; T2, N1, M0) was a carrier of the BRCA-1 mutation but had no family history of BC. In over 50% of cases, there was a negative family history of BC. However, no group comparisons were performed regarding this risk factor and tumor signature. Knowing the family history of BC at an early age is important, because the adjusted relative risks of developing the disease among young offspring aged less than 35 years is higher than among those aged 45–54 years, with a RR = 3.22 and RR = 1.51, respectively [25]. In addition, 50% of women with a positive family history that develop BC before the age of 30 are carriers of BRCA-1, BRCA-2, or TP53 mutations [24]. BRCA-1 and -2 mutations, in the mother or sisters, increase the risk for breast (RR = 6.3) and ovarian cancer (RR = 5.3) in young women, but, within mutation-positive families, the risk of BC increases 26.6-fold [26]. In this cohort, BRCA mutations were detected in only 10% of all patients, similarly as in the literature [27].

Another known risk factor for BC is the use of hormonal contraceptives; however, in this cohort, it was not possible to determine the time of use and type of contraceptive. Therefore, this risk factor was not analyzed.

In reference to obesity, 10 of patients (11.3%) presented with a BMI ≥ 30 kg/m^2^; four of them had recurrence, and one died during the follow-up period. Recent studies have shown that obesity is variably associated with the risk of developing BC and poorer oncological outcomes [28]. A meta-analysis of preventable factors regarding the risk of BC incidence, conducted by Poorolajal J et al. [29] and including 19,413,702 participants from prospective cohort studies, shows that the relative risk of BC development is increased in overweight and obesity postmenopausal women (RR = 1.18; 95% CI: 1.13 to 1.24) but not in premenopausal women (RR 0.92; 95% CI: 0.82 to 1.03). The results from the meta-analysis conducted by Lohmann AE et al. [30] show that, in comparison to non-obese women, obesity at the time of BC diagnosis negatively affects the disease-free survival period (DFS) in all BC subtypes, being worse for HR + HER2- cases (HR = 1.26; 95% CI: 1.13 to 1.41), and it significantly reduces the overall survival (OS) of patients exhibiting cancer subtypes HR + HER2- (HR = 1.39, 95% CI: 1.20 to 1.62), HER2+ (HR = 1.18; 95% CI: 1.05 to 1.33), and TNBC (HR = 1.32; 95% CI: 1.13 to 1.53). These negative effects on oncological outcomes are associated with therapy resistance in tumor cells caused by the alteration of extracellular matrix remodeling and adipocyte function, which lead to the increased local secretion of cytokines, adipokines, and estrogen [30].

In our hospital, all patients undergo guideline-based therapy after interdisciplinary consultation among the tumor board prior to the initiation of the first therapy, in case of disease progression or relapse. Accordingly, 23.9% of cases were treated neoadjuvantly and two thirds of patients required adjuvant therapies. Breast-conserving surgery with an oncoplastic approach is usually preferred; in one quarter of the entire cohort, a mastectomy was performed. After surgery, 60.2% of cases experienced mild seroma and hematoma, corresponding to complications of Clavien–Dindo grade I; only one patient required unilateral breast revision due to hematoma after a bilateral mastectomy. Other studies have reported similar frequencies, with seroma formation being the most common postoperative complication, occurring in up to 85% of cases. This is significantly more frequent after a mastectomy than after breast-conserving surgery [31], but no significant difference in complication rates has been found between conservative surgery with and without oncoplastic procedures [32,33,34,35].

The risk of local recurrence after conservative surgery is nine-fold higher among women aged <35 years that present with even early-stage tumors [23]. These techniques are especially challenging for patients with small- to medium-volume breasts because, after the removal of the tumor, the remaining glandular tissue is often insufficient to completely fill the defect [36,37,38]. Nevertheless, better esthetic results are usually achieved after oncoplastic approaches [36,37,38,39,40], but there is a paucity of information on the long-term oncological outcomes of these new reconstructive approaches [35,36,37,38,40].

Regarding adverse events after chemo- and radiotherapy, most of the patients presented mild toxicity, and SAE grade 3 were reported in 15.9% of cases. These complication rates are similar to worldwide reports, showing that healthy young women and those exhibiting early-stage BC usually present fewer treatment-related complications [41]. In a recent multivariate analysis regarding the treatment outcomes and underlying health statuses of 2000 US women, Ong et al. [42] found that younger women were generally healthier and had fewer comorbidities at diagnosis and thus had significantly lower risks of complications during the first year of multimodal therapy. The opposite was observed among women with poor general health, mainly in those older than 60 years and with underlying congestive heart failure. This high-risk group is significantly more likely to experience more than three treatment-related AEs or death than those specifically associated with BC. The authors concluded that the negative impact of comorbidities on treatment outcomes might be due to the effects of interventions and the toxicity of chemotherapeutic agents on the natural course of the preexisting diseases themselves; the evidence regarding the association between comorbidities and readmission is scant.

Women experiencing AEs are also susceptible to readmission. Miret et al. [43] reported that, among 1055 Spanish women who underwent BC screening and treatment, 7.2% required readmission in the first month (early), 8.2% in the first year (late), and 6.7% occurred >1 year (long-term) after the first treatment. Compared to cases with no readmission, early readmissions were significantly more common in patients younger than 60 years (64.5%); the rates of multifocal and moderately differentiated tumors were significantly higher in these women. Luminal A tumors were diagnosed more frequently in the early and late readmission groups, while the frequency of HER2 tumors was higher in women with prolonged readmissions. Re-excision and mastectomy or lymphadenectomy were the most frequent causes of early readmission (80%), while disease progression was the most frequent cause of long-term readmission (78.9%). A higher risk of early readmission was seen in patients that experienced a surgical complication (aOR = 3.62; 95% CI: 1.27 to 10.29), mainly wound disturbances and surgical-site infections. Meanwhile, medical complications were significantly associated with the risk of late (aOR = 8.72; 95% CI: 2.83 to 26.86) and long-term readmission (aOR = 4.79; 95% CI: 1.41 to 16.31).

In this series, only patients with SAE were readmitted, but they did not require intensive medical care or further surgical treatment; they required a modification in their treatment regimen, but none of them died due to complications. Data from a retrospective analysis regarding the impact of chemotherapy schedule modification on the oncological outcomes of 171 BC patients [44] showed that almost 70% of cycle schedule modifications occurred for medical reasons and the other 30% because of a request by the patient or an administrative cause. The authors report that cumulative delays of ≥14 days have statistically significant, negative effects on patient survival, increasing the risk of death by 2.56 times for patients with a schedule modification (95% CI: 1.10 to 5.99; *p* = 0.030), and there is a 3.44-times higher risk for patients with an incomplete schedule (95% CI: 1.32 to 9.03; *p* = 0.012) in comparison to patients with no schedule modifications.

During the longitudinal patient follow-up period, most cases remained disease-free, with 3- and 5-year recurrence-free survival rates of 84.9% and 77.3%, respectively. The comparative analysis between the groups showed that most recurrences occurred locally, after the completion of the initial curative treatment, and in the TNBC and luminal B HER2-negative groups. The groups with the fewest recurrences were the luminal A and Ki67-unknown groups. The patient with a adenoid cystic carcinoma remained recurrence-free during the observation period. These results are similar to other reports showing that the use of neo- and adjuvant therapies in young BC patients reduces the risk of relapse in 35% of ER- and in 50% of ER+ subtypes [23].

Recently, transnational studies have explained the genetic and molecular alterations that occur within tumor cells, which explain why some patients remain free of relapse while other patients present resistance to treatment, progressive disease, or cancer recurrence. Beyond tumor heterogeneity, understood as differences in the molecular subtypes of BC according to hormone receptors, intratumoral heterogeneity has been observed [45]. This heterogeneity refers to the coexistence of tumoral cells of different phenotypes (clonal heterogeneity) and in different cell states (cell-state heterogeneity) within a single tumor. To illustrate this, different areas of the same tumor can express ER, PR, and HER2 at different levels according to immunohistochemistry. In TNBC tumors, adaptive genomic selection and transcriptional reprogramming processes, characterized by glycosphingolipid metabolism and lysosomal turnover, affect the cytokine pathways involved in the innate immune responses of tumor cells and lead to the selection of chemoresistant tumor cell subpopulations. Intratumoral heterogeneity can also explain the ER, PR, and HER2 receptor expression conversion of metastatic lesions in HER2-negative primary tumors [45], as well as the aggressive nature of some tumors. In addition to this, gene expression in normal mammary tissue and tumors might be influenced by the menstrual cycle, giving rise to the unique biology of BC in premenopausal women [41]. A better understanding of these mechanisms will allow the identification of new targets for BC therapeutic strategies.

Regarding survival in the present analysis, among the entire study population, 92.1% were followed for more than 3 years. The 3- and 5-year survival rates were 93.1% and 90.3%, respectively. Ten patients died during their follow-up periods, mainly during the first three years of treatment. Half of the deceased patients had TNBC, three had luminal B HER2-negative tumors, one had a HER2-positive tumor, and one had a luminal B HER2-positive tumor. There were no deaths in either the luminal A or Ki67-unknown groups. Patients with luminal B HER2-positive and luminal B HER2-negative tumors had slightly lower 5-year survival rates, at 92.9% and 88.4%, respectively, and those with TNBC had the lowest survival rate at the 5th year (77.3%). Different reports show that an age younger than 35 is a strong prognostic factor for overall mortality, with these patients being 44% to 50% more likely to die in comparison to women older than 40 years [41]. The risk of BC-related death is higher in young women diagnosed with TNBC (HR = 2.7) and hormone-receptor-negative HER2+ tumors (HR = 1.6). Mutations in the TP53 gene are more common in young women, increasing the risk of death by 2.27%, particularly in patients with hormone-receptor-negative disease [23].

Further log-rank tests showed no statistically significant differences in overall survival in this cohort according to the tumor subtype (*p* = 0.164) or chemotherapy regimen (*p* = 0.362), but showed that an advanced tumor stage correlated statistically significantly with worse overall survival (*p* < 0.001). Advanced-stage disease at diagnosis negatively affects the prognosis and survival rates. In comparison to these results, a larger study involving 1228 patients from India reported a 5-year OS rate of less than 79.6%, with patients with advanced stage III disease having the lowest DFS (54.4%) and OS (81.8%) rates [46]. Furthermore, the low mortality rate observed in the present cohort reflects the positive impact of the availability of resources for individualized and targeted therapy. An analysis of the BC-related mortality rates in Germany indicates a trend towards a reduction in this outcome (−12.9%), although the rates are still stable within younger women, being −0.8% in those aged 20 to 39 years and 0.2% in those aged 40 to 49 years, but increased in those aged 70 to 79 (+0.7%) and >80 years (+2.9%) [24]. This trend reflects the positive impact of screening programs, improved diagnostic techniques, and therapy compliance and the benefits of guidelines and the implementation of certified reference centers for women affected by and living with BC [24,44,47,48]. However, there is a lack of harmonization within European guidelines [48], and the impact of management guidelines on oncological outcomes differs depending on the availability of local resources for its implementation in daily practice. In addition, an American analysis of the impact of centralized oncology care [49] demonstrated that this modality introduces disparities for some subsets of the population and a significant access barrier due to travel requirements and other sociodemographic factors, which limit improvements in oncological outcomes.

Like other retrospective studies, this analysis was subject to certain limitations due to data availability, particularly with regard to BC risk factors other than age and the treatment regimen. Furthermore, this was a single-institution study, which may restrict its generalizability. Another limitation of this study is the lengthy period during which the different therapeutic approaches were employed, spanning almost one decade. Nevertheless, given the relatively low incidence of this cancer in this age group, comprising only 4% of all breast cancers, and the fact that these patients are usually underrepresented in clinical trials, we present the outcomes of patients with bilateral cancer and metastatic breast cancers (MBCs). These data contribute to the existing international evidence and can be used in further meta-analyses and reviews. Further scientific research is needed to investigate the role of CDK4/6 inhibitors in high-risk ER(+) patients, the addition of pertuzumab and TDM-1 in HER2-enriched subsets, and immunotherapy in the neoadjuvant treatment of BC patients.

## 5. Conclusions

The results of this analysis show once again that women who present with BC before the age of 40 tend to have fast-growing and triple-negative carcinomas. The majority of the patients were in good health, presented with localized disease, and adhered well to the recommended therapies. Most recurrences occurred locally, after the completion of the initial curative treatment, especially in patients diagnosed with TNBC and HER2-positive (non-luminal) tumors. Deaths occurred mainly during the first three years of treatment, mostly in the TNBC group. Although no statistically significant differences in overall survival were observed according to the tumor biology, the trend was still indicative of a potential difference. In accordance with the findings of previous studies, this analysis demonstrated that TNBC is the most frequently occurring tumor type in young women and is associated with a worse prognosis. More research is needed on the pathomechanisms of BC development in young women, especially those leading to disease progression and resistance to therapy. A better understanding of these mechanisms will allow the identification of new targets for BC therapeutic strategies.

## Figures and Tables

**Table 1:** Demographic characteristics of women diagnosed with breast cancer before the age of 40 years.

		n = 88	Frequency
Age (years)	20–24	1	1.1%
	25–29	8	9.1%
	30–34	25	28.4%
	35–39	54	61.4%
Gravidity	0	28	31.8%
	1	21	23.9%
	2	26	29.5%
	3	7	8.0%
	4	6	6.8%
BMI (kg/m^2^)	Low	2	2.3%
	Normal	50	56.8%
	Overweight	26	29.5%
	Obesity I	8	9.1%
	Obesity II	1	1.1%
	Obesity III	1	1.1%
Family history of breast cancer	Yes	38	43.2%
	No	49	55.7%
	Unknown	1	1.1%
BRCA mutation	Yes	9	10.2%
	No	12	13.6%
	Unknown	67	76.2%

**Table 2:** Characteristics of all breast cancers diagnosed in a cohort of women under the age of 40 years.

**(a) Tumor Stage**		Tumors * (n = 90)	**Frequency**
Tumor size	T1a	2	2.2%
	T1b	11	12.2%
	T1c	43	47.8%
	T2	31	34.5%
	T3	3	3.3%
	T4	0	0.0%
Lymph node metastasis	N0	56	62.2%
	N1	23	25.6%
	N2	7	7.8%
	N3	4	4.4%
Distant metastasis	M0	87	96.7%
	M1	3	3.3%
Lymph vessel invasion	L0	51	56.7%
	L1	25	27.8%
	Not described	14	15.5%
Vascular vessel invasion	V0	73	81.2%
	V1	3	3.3%
	Not described	14	15.5%
**(b) Tumor Characteristics**		n = 90 *	**Frequency**
Histology	Non-specific type	87	96.7%
	Invasive lobular	1	1.1%
	Invasive mucinous	1	1.1%
	Adenoid–cystic	1	1.1%
Subtype	Luminal A	10	11.1%
	Luminal B HER2-negative	21	23.3%
	Luminal B HER2-positive	14	15.6%
	HER2-positive	6	6.7%
	Triple-negative	24	26.7%
Nuclear grade	G1	2	2.2%
	G2	29	32.2%
	G3	58	64.4%
	Grading not reported	1	1.1%
Ki67 rate	≤14%	11	12.2%
	>14%	57	63.3%
	Not reported	22	24.5%
Receptor status	Estrogen-positive	59	65.5%
	Estrogen-negative	31	34.5%
	Progesterone-positive	56	62.2%
	Progesterone-negative	34	37.8%
	HER2neu-status-positive	22	24.4%
	HER2neu-status-negative	68	75.6%

* The denominator for this analysis was the total number of tumors diagnosed in the entire study population. (a) Tumor stage; (b) Tumor characteristics.

## Data Availability

As the database contains personal and clinically sensitive data, it is not possible to share it. Therefore, the raw data supporting the conclusions of this article will only be made available by the authors upon request. Research applications are reviewed by qualified scientific experts and the data protection officer. The applicant will then be asked to sign a data sharing agreement to ensure the protection of patient confidentiality and any intellectual property rights prior to the release of data.
